# Design and performance of GaSb-based quantum cascade detectors

**DOI:** 10.1515/nanoph-2023-0702

**Published:** 2024-01-18

**Authors:** Miriam Giparakis, Andreas Windischhofer, Stefania Isceri, Werner Schrenk, Benedikt Schwarz, Gottfried Strasser, Aaron Maxwell Andrews

**Affiliations:** Institute of Solid State Electronics, TU Wien, Gußhausstraße 25, 1040 Vienna, Austria; Center for Micro- and Nanostructures, TU Wien, Gußhausstraße 25, 1040 Vienna, Austria

**Keywords:** quantum cascade detector, mid-infrared detection, molecular beam epitaxy, III–V semiconductors, InAs/AlSb on GaSb

## Abstract

InAs/AlSb quantum cascade detectors (QCDs) grown strain-balanced on GaSb substrates are presented. This material system offers intrinsic performance-improving properties, like a low effective electron mass of the well material of 0.026 *m*_0_, enhancing the optical transition strength, and a high conduction band offset of 2.28 eV, reducing the noise and allowing for high optical transition energies. InAs and AlSb strain balance each other on GaSb with an InAs:AlSb ratio of 0.96:1. To regain the freedom of a lattice-matched material system regarding the optimization of a QCD design, submonolayer InSb layers are introduced. With strain engineering, four different active regions between 3.65 and 5.5 µm were designed with InAs:AlSb thickness ratios of up to 2.8:1, and subsequently grown and characterized. This includes an optimized QCD design at 4.3 µm, with a room-temperature peak responsivity of 26.12 mA/W and a detectivity of 1.41 × 10^8^ Jones. Additionally, all QCD designs exhibit higher-energy interband signals in the mid- to near-infrared, stemming from the InAs/AlSb type-II alignment and the narrow InAs band gap.

## 1 Introduction

Mid-infrared (MIR) photonics is positioned as an important technology with applications in spectroscopy [1, 2], medicine [3], security [4], imaging [5], and free space optical telecommunication [6, 7]. Quantum cascade detectors (QCDs) are zero-bias MIR detectors composed of semiconductor heterostructures with alternating well and barrier material. The desired absorption wavelength is tailorable over a wide range by changing the well and barrier thicknesses of the heterostructure, and thus adjusting the discrete energy levels. The primary performance metrics of detectors, like QCDs, are the responsivity and detectivity. QCDs stand out, due to a narrowband (typically ≤0.08 eV [8]) spectral response and high-speed detection [9] resulting from the sub-picosecond intersubband unipolar transitions [10–12]. QCDs are photovoltaic in nature and therefore exhibit low-noise and room-temperature operation. This contrasts with the competing intersubband technology quantum well infrared photodetectors (QWIPs) [13], which have a significant temperature-dependent dark current, stemming from the applied bias, reducing the detectivity. At cryogenic temperatures, QWIPs offer higher responsivity compared to standard 45°-facet double-pass geometry QCDs [14–16]. However, single-period QCDs [7, 17] have a comparable responsivity. Interband detectors, like type-II and other superlattice detectors, offer a fundamentally different working principle. These detectors operate in the visible to MIR range with high responsivity [18, 19] and a broadband response of around 2 eV. In comparison, QCDs are preferable for high-speed operation, narrowband, and longer wavelength photodetection.

While both, the QCD detectivity and responsivity can be optimized through the design, they can also be improved by material-intrinsic parameters, like the effective electron mass $m_{e}^{*}$ of the well material. A low $m_{e}^{*}$ increases the optical transition strength, improving the responsivity. Additionally, a low $m_{e}^{*}$ leads to increased detectivity, because of the reduced scattering rates, and therefore lower noise. The second important intrinsic material system property is the electron barrier height, known as the conduction band offset (CBO). The CBO limits the shortest detectable wavelength for optical intersubband transitions. In the most widely used material system for QCDs, In_0.53_Ga_0.47_As/In_0.52_Al_0.48_As lattice matched to InP substrates [20], the CBO is 0.52 eV [21], which can be extended by strain engineering up to 0.61 eV [22]. This CBO limits QCDs grown with this material system to detectable wavelengths ≳3 µm. To extend QCDs to shorter wavelengths, material systems with higher CBOs are required, which are beneficial for higher resistance, ultimately increasing the detectivity.

A promising material system having both, one of the lowest $m_{e}^{*}$ of 0.026 *m*_0_ [21] and one of the highest CBOs of 2.28 eV [21] is InAs/Al(As)Sb, which is either grown lattice matched to InAs substrates [8, 23] or strain balanced to GaSb substrates [24].

## 2 Materials and methods

### 2.1 Principles and growth analysis

Due to the intersubband character of QCDs, they are only sensitive to illumination polarized parallel to the growth direction (out-of-plane polarization) [25]. In contrast to the 300 K band gap of 0.36 eV [21] of InAs substrates, the larger 0.73 eV [21] band gap of GaSb allows for illumination through the substrate in the standard 45°-facet double-pass method for wavelengths ≳1.7 µm, compared to 3.44 µm for InAs. On GaSb substrates, InAs layers have a tensile mismatch of 0.62 %, and AlSb layers have a compressive mismatch of −0.64 %. The critical layer thickness on GaSb, calculated with the formula of Matthews and Blakeslee’s [26], is for both materials approximately 35 nm. A strain-compensated growth of InAs/AlSb on GaSb is therefore achieved with an InAs:AlSb layer-thickness-ratio of 0.96:1, which would result in approximately equally thick barriers and wells. Considering the necessary InAs well thicknesses of a QCD design for 4.3 µm is between 2.95 and 7.2 nm, equal-thickness AlSb barriers would drastically reduce the QCD’s figures of merit, due to an insufficient extraction efficiency. To optimize a QCD design, it is necessary to change the well and barrier thicknesses independently of each other, especially because of the high CBO, the AlSb barriers need to be thinner than conventional designs. One trick to achieving this lies in designing the interfaces. Since InAs and AlSb do not have any constituents in common, one of two interfaces can form at the interface between the well and barrier, either InSb or AlAs. To achieve strain-balancing with decreasd AlSb barrier thickness, one needs a material with a compressive mismatch to GaSb, which is given for InSb with a −5.92 % mismatch. Previous studies on InAs/AlSb superlattices found increased carrier mobility for InSb interfaces compared to AlAs interfaces [27], thus making them the preferred interfaces. Due to the high mismatch of InSb to GaSb, submonolayer thick InSb layers are sufficient to strain-balance an optimized InAs/AlSb QCD design, with the advantage that such thin InSb layers do not influence the band structure design. For the QCD growth, the submonolayer thick InSb layers were first calculated and then experimentally calibrated by strain-balanced test structures. The process proved itself to be reproducible, as determined by HR-XRD. To achieve sharp interfaces and limit the As-for-Sb exchange [28], shutter sequences and shutter opening times were developed similarly to Refs. [27, 29, 30].

**Figure 1:**
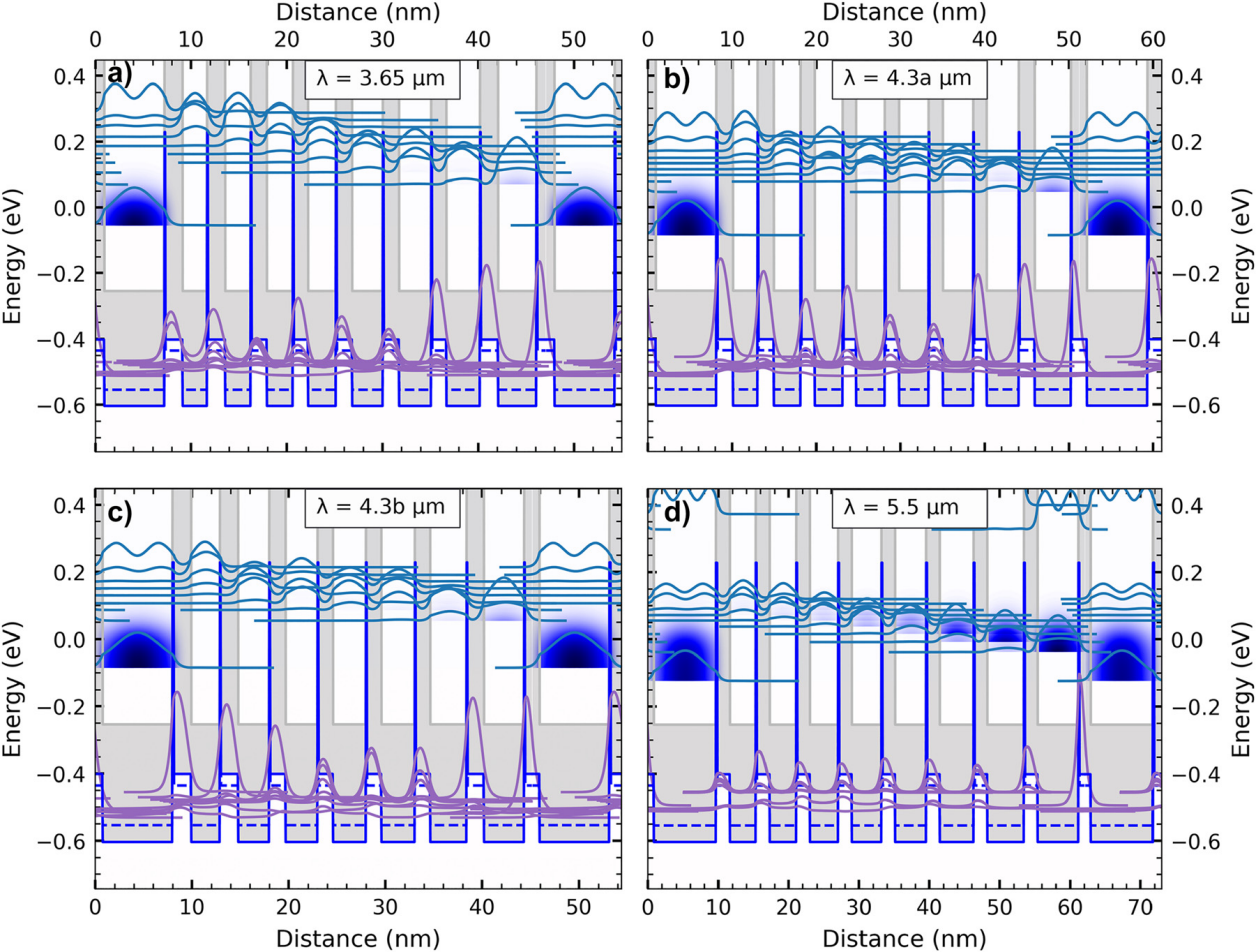
Band diagrams for the four designs from 3.65 to 5.5 µm. Shown are the probability densities of the energy levels, where the first well corresponds to the optical transition well. The blue contour, mainly in the ground state of the optical well, corresponds to the electron occupancy. The active region was repeated 20 times for all devices. The conduction band edge is dark gray, the valence band is blue, the dashed blue line is the light-hole valence band, and the solid blue line is the heavy-hole valence band of the InAs/AlSb material system, the band gap is light gray. The blue peaks reaching into the InAs conduction band are the submonolayer InSb layers valence bands. (a) 3.65 µm peak responsivity with the layer sequence in nm: **1.80**, 6.30, **1.80**, 2.55, **1.80**, 2.65, **1.60**, 2.70, **1.50**, 2.90, **1.60**, 3.20, **1.60**, 3.35, **1.50**, 3.50, **1.80**, and 4.0. (b) 4.3 µm peak responsivity (design 4.3a) with the layer sequence in nm: **1.80**, 7.20, **1.80**, 2.95, **1.80**, 3.20, **1.60**, 3.30, **1.50**, 3.40, **1.60**, 3.50, **1.60**, 3.60, **1.50**, 3.80, **1.80**, and 4.30. (c) 4.3 µm peak responsivity (design 4.3b) with the layer sequence in nm: **1.50**, 7.20, **1.80**, 2.95, **1.80**, 3.20, **1.60**, 3.30, **1.50**, 3.40, **1.50**, 3.45, **1.50**, 3.75, **1.70**, and 4.15. (d) 5.5 µm peak responsivity with the layer sequence in nm: **1.60**, 8.85, **1.80**, 3.70, **1.80**, 3.80, **1.80**, 4.0, **1.80**, 4.20, **1.80**, 4.40, **1.80**, 4.80, **1.80**, 5.20, **1.80**, and 5.80. The boldly printed layers are the AlSb barriers, and the underlined well is Te doped 8 × 10^17^ cm^−3^.

In the following, the results of the four InAs/AlSb QCD designs on GaSb ranging from 3.65 to 5.5 µm are presented. The QCDs are designed using an eight-band k⋅p-method, including scattering mechanisms such as longitudinal optical (LO) and acoustic phonon scattering, as well as interface roughness scattering, and alloy scattering. Figure 1 shows the active region band structure design for each QCD, where (a) is the 3.65 µm design, (b) the first design for 4.3 µm, referred to as the 4.3a µm design, (c) the second design for 4.3 µm, referred to as the 4.3b μm design, and (d) the 5.5 µm design. The conduction band edge is depicted in dark gray, and the valence band edge is in blue. The designs are based on a vertical optical transition. The optical well is the first well of each structure. The blue contour of the probability density, mainly in the first well, corresponds to the electron occupation. Once the transition occurs due to photon absorption, the electrons are extracted from the first excited state in the first well, which is nearly resonant to the first state of the extraction region. The energy separation of the next states and the thin barriers allow for sub-picosecond scattering times along the extractor ladder. To ensure reduced thermal backfilling, the lowest extractor state has a higher energy separation from the ground state of the next optical transition. This increases the electron lifetime in the ground level of the optical well and benefits the absorption efficiency. Design 4.3b μm corresponds to a refined version of design 4.3a µm with thinner barriers and a higher energy separation of the last extractor state to the next optical ground state, which allows for reduction of the active region by one well and barrier in the extractor, increasing the extraction efficiency. The blue lines extending into the InAs conduction band stem from the valence band of the submonolayer thick InSb strain-balancing layers. The layers are too thin to allow states in the InSb to form. The areas drawn in light gray correspond to the band gap. Due to the type-II band alignment of InAs/AlSb and the small band gap of InAs, interband transitions in the MIR to near-infrared (NIR) are expected.

The four QCDs were grown by molecular beam epitaxy with a Riber Compact 21 and are doped with Te, since Si exhibits strong amphoteric behavior in Sb-compounds. Therefore, Te avoids unwanted p-type doping in AlSb barriers that could result from Si dopant diffusion into the barriers. The active regions consist of 20 periods grown between 200 nm-thick short-period InAs/AlSb superlattice contacts, where the InAs wells are Te doped to 3 × 10^18^ cm^−3^. Additional transition layers from the GaSb substrate to the superlattice contact and from the superlattice contact to the active region were necessary for the final device design.

**Figure 2:**
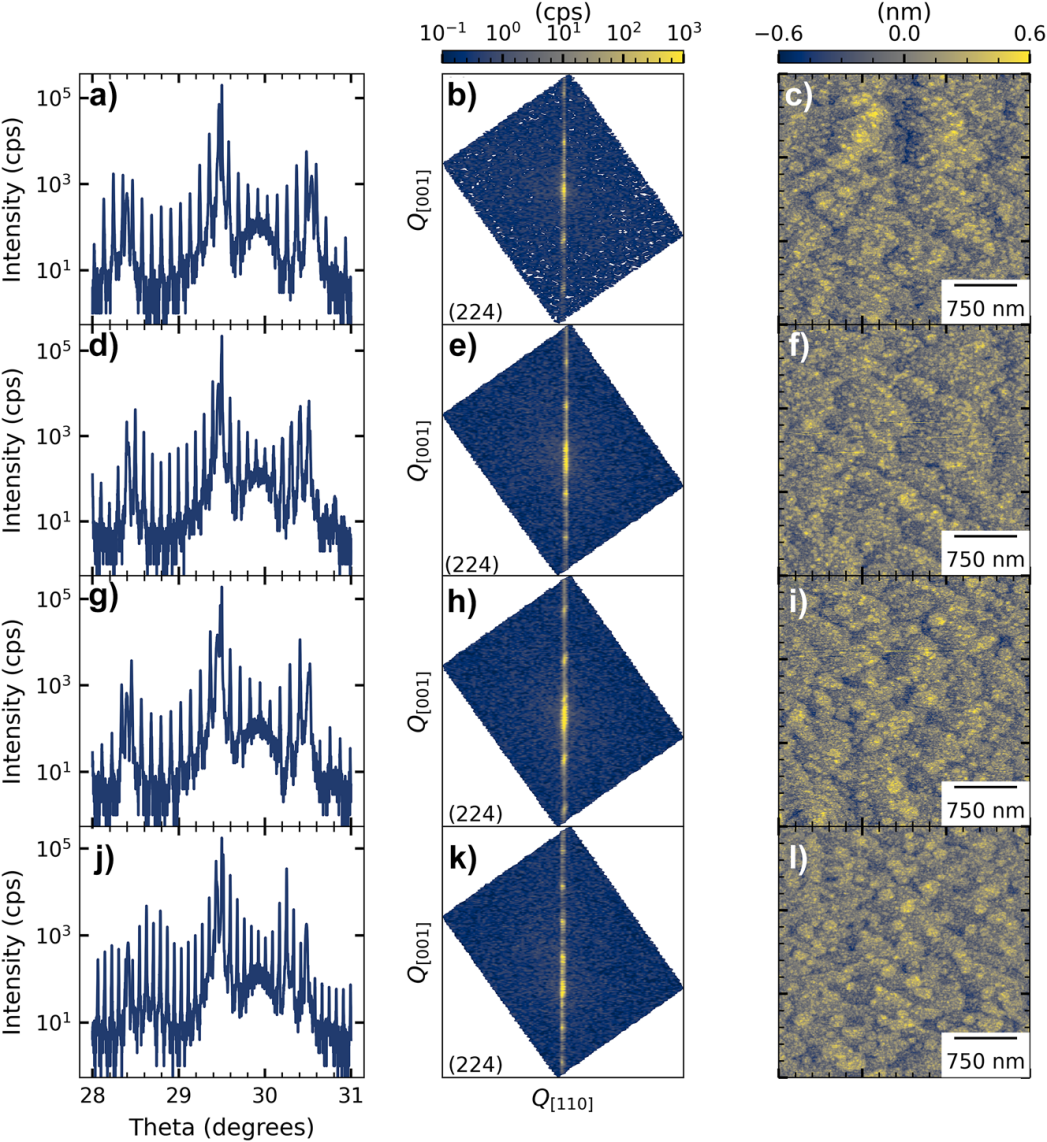
Growth analysis of the InAs/AlSb QCDs on GaSb of (a), (b), & (c) design 3.65 µm, (d), (e), & (f) design 4.3a µm, (g), (h), (i) design 4.3b μm, and (j), (k), & (l) design 5.5 µm: left: HR-XRD (004) *ω*-2*θ* scans, middle: HR-XRD 0.5° × 0.5° (224) reciprocal space maps, and right: 3 × 3 µm AFM scans with a root-mean-square surface roughness of (c) 0.188 nm, (f) 0.171 nm, (i) 0.197 nm, and (l) 0.165 nm.

Figure 2 shows the growth analysis of all QCDs, where (a–c) correspond to the 3.65 µm design, (d–f) to the 4.3a µm design, (g–i) to the 4.3b μm design, and (j–l) to the 5.5 µm design. The growth was analyzed using three methods: High-resolution X-ray diffraction (HR-XRD) *ω*-2*θ* scans with a Philips MRD Pro, left column, HR-XRD (224) reciprocal space maps, middle column, and atomic force microscopy (AFM) with a Veeco Dimension V, right column.

As observed in the HR-XRD *ω*-2*θ* scans, the highest intensity peak is the substrate followed by the zeroth-order active region peak, where good strain balancing is observed for all QCDs. The third peak to the left corresponds to the zeroth-order short-period contact superlattice peak. The modulation between 29.6 and 30.0° stems from a 20 nm-thick highly-doped InAs layer top contact. The sharp peaks observed in all scans indicate low interface roughness. The (224) reciprocal space maps of the QCDs show a perfectly vertical intensity contour, which indicates a fully strained material growth. From the intensity maxima, no increased mosaic spread (which shows high crystal quality), interface roughness, or growth rate fluctuations are apparent, which would result in horizontal or vertical broadening of higher-order peaks [31]. AFM scans of a 3 × 3 µm surface of the QCDs confirm the low interface roughness with low root-mean-square surface roughness values of 0.188 nm for the 3.65 µm design, 0.171 nm for the 4.3a µm design, 0.197 nm for the 4.3b μm design, and 0.165 nm for the 5.5 µm design.

### 2.2 Fabrication and optical characterization

**Figure 3:**
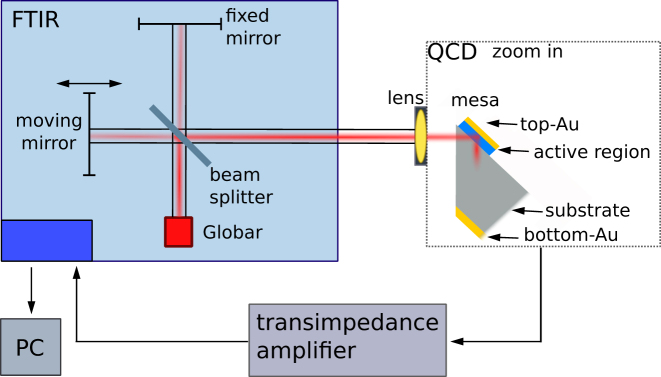
Schematic measurement setup for responsivity measurements of QCDs. The measurements were performed using an FTIR with a MIR Globar source. The QCDs were fabricated into the 45°-facet double-pass configuration. The current output of the QCDs is fed as an analog voltage signal back to the FTIR by the transimpedance amplifier. For mixed (both)/in-plane polarization measurements, a MIR polarizer is placed before the lens.

The QCDs were fabricated into the 45°-facet double-pass geometry, a standard structure for intersubband device characterization, which allows a comparison to QCD devices in literature [10]. Figure 3 depicts a sketch of the measurement setup, including the fabricated QCD and its light-coupling mechanism. For this configuration, a facet under an angle of 45° is polished substrate-side onto the wafer edge of the QCD. The device is then illuminated through the substrate, with the facet oriented normal to the light source. Due to the intersubband selection rule, the QCD structure is only sensitive to light polarized out-of-plane, with respect to the quantum well. However, due to the 45° measurement geometry of the QCD, out-of-plane polarized light is coupled in at an angle of 45° and therefore still contains both polarizations (mixed).

The QCDs are fabricated into 150 × 150 µm mesas, which were defined by a Cl/Ar dry etching process using an inductively coupled plasma (ICP) process, resulting in smooth, slightly positively sloped sidewalls. The top contact (10/380 nm Ti/Au) is on top of the mesa, and the bottom contact (10/380 nm Ti/Au) is on the backside of the *n*-type 1–4 × 10^17^ cm^−3^ doped GaSb substrate.

Next, the QCDs were optically characterized with a Bruker Vertex 70v Fourier-transform infrared spectrometer (FTIR) with a Globar as a broadband unpolarized light source and a Thorlabs PDA200C transimpedance amplifier to obtain the responsivity and detectivity. For this, the following steps were performed at ambient conditions [32]:

The spectrum of the Globar, its beam spot profile, and the power of the entire beam spot were recorded in the measurement configuration with an Ophir Laserstar detector. As the Globar beam spot is significantly larger than the QCD mesa, only the incident light intensity *P*_*ω*_ on the mesa area was considered, using the highest intensity area of the beam spot. For each QCD, the spectrum of 3–4 mesas was recorded under the same conditions, and current–voltage (I–V) characteristics in aligned and dark conditions were measured with a Keithley 2612B. To obtain the responsivity, the photocurrent *I*_*p*_ was extracted from the aligned I–V measurements at 0 V, where the dark current at 0 V was subtracted.

The responsivity spectrum *R*_*p*_ is obtained by

(1) $$R_{p}=\frac{I\left(f\right)}{P\left(f\right)}$$

where *I*(*f*) is the spectrally resolved photocurrent, with *I*_*p*_ = ∫*I*(*f*)d*f*, and *P*(*f*) is the spectrally resolved Globar incident light intensity with *P*_*ω*_ = ∫*P*(*f*)d*f*. The specific Johnson noise limited detectivity *D* was obtained using [10]:

(2) $$D=R_{p}\sqrt{\frac{R_{0}A}{4k_{B}T}},$$

where *R*_0_ *A* is the differential resistance optical area product of the device at zero bias, *k*_*B*_ is the Boltzmann constant, and *T* is the temperature.

## 3 Results and discussion

**Figure 4:**
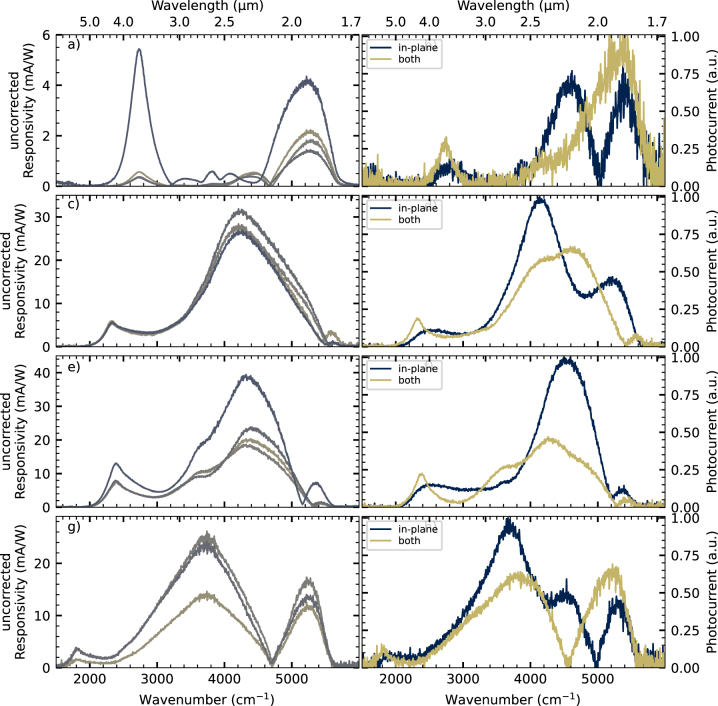
Responsivity measurements for the four QCDs. Left: uncorrected responsivity of each QCD design. The multiple lines per plot correspond to multiple mesas measured. Right: normalized out-of-plane polarization and in-plane polarization measurements for a single mesa. Design 3.65 µm is shown in (a) & (b), 4.3a µm is shown in (c) & (d), 4.3b μm is shown in (e) & (f), and 5.5 µm is shown in (g) & (h).

The left column in Figure 4 shows the uncorrected responsivity spectra of the QCDs. Since a Globar is an unpolarized source and due to the measurement in the 45°-facet double-pass configuration, the QCD responsivity is usually corrected by taking just out-of-plane polarization into account with a multiplication factor of two of the uncorrected/unpolarized responsivity. A responsivity peak at the designed QCD detection wavelength is observed for all QCDs. The device-to-device reproducibility strongly depends on the facet quality over the length of the sample piece, where the mesas were processed in a row. With the multiplication factor as a correction, the peak room-temperature responsivities at the QCD transitions are: 10.85 mA/W and 1.17 × 10^8^ Jones for the 3.65 µm design, 12.5 mA/W and 3.81 × 10^7^ Jones for the 4.3a µm design, 26.12 mA/W and 1.41 × 10^8^ Jones for the 4.3b μm design, and 8.3 mA/W and 1.73 × 10^7^ Jones for the 5.5 µm design. A broadband interband signal at shorter wavelengths is observed in Figure 4 in all spectra, with a cut-off caused by the GaSb substrate, its origin is explained below.

The right column in Figure 4 shows the mixed (both) and in-plane polarization-dependent measurements. It is expected that the responsivity at the designed QCD wavelength is only due to out-of-plane polarization. However, the in-plane polarization signal at the designed QCD transition is not vanishing, which would be expected from the intersubband selection rule. Since the in-plane polarization signal is at the QCD signal for all four different QCD designs with different transition energies, the interband transition causing the in-plane polarization signal is expected to be QCD design dependent, but there are no interband transitions in the band structure corresponding to the designed optical transition energy. Currently, the origin of these in-plane polarization signals is unclear, but it may be due to polarization mixing due to the lens after the polarizer or irregularities in the polished 45°-facet.

A polarization dependency is also observed for the broadband interband signal at shorter wavelengths. This polarization dependency of interband transitions can stem from multiple parameters:

– The valence band is formed of bands exhibiting non-negligible nonparabolicity, due to their p-band character [33], which is particularly strong in narrowband materials such as InAs. This contrasts with the conduction band. The resulting transition energy therefore depends on the optical matrix element between the subbands. The transition can occur at non-zero *k* values and can be different from the energy at the Gamma point.
– Heavy- and light-holes have different absorption polarization dependencies [34], although there is not such a prominent difference as in intersubband transitions.
– Valence band mixing at a non-zero wave vector *k* occurs, where states now have a mixed heavy- and light-hole character [35].

**Figure 5:**
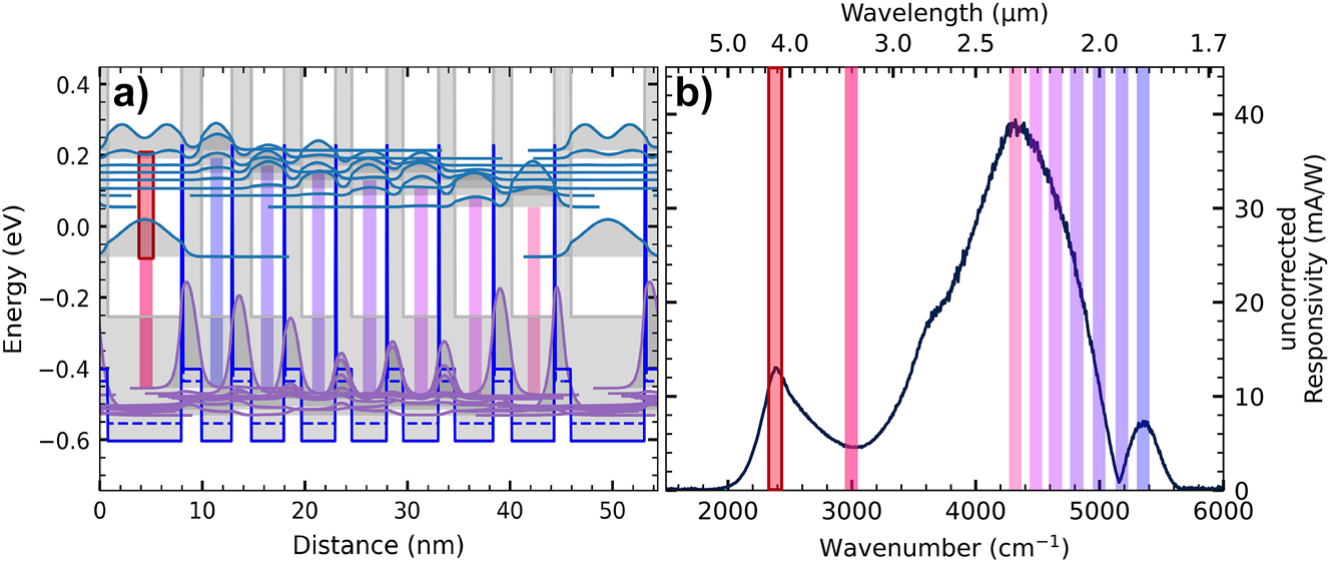
Comparison of the electron transitions in the band structure design with the responsivity measurement. (a) Band structure with probability densities of design 4.3b μm. The optical transition is highlighted in red. Other colors depict possible interband transitions from states in the AlSb valence band. (b) Uncorrected responsivity measurement of design 4.3b μm. The highlighted areas correspond to the transition energies highlighted in the same color in (a).

In the following, the origin of the broadband interband signal at energies higher than the QCD signal is discussed for the 4.3b μm design, with the help of Figure 5. As mentioned, MIR to NIR interband transitions from the AlSb valence band to the states of the InAs conduction band are possible. In Figure 5(a) the optical intersubband transition is highlighted in red in the first well. The other colored lines indicate the corresponding interband transitions, with the lowest energy from the AlSb valence band to the ground state of the optical transition well. The highest transition energy occurs from the AlSb valence band to the highest ground state in the extractor, which also matches a possible interband transition in the superlattice contact. Figure 5(b) shows the possible transitions in comparison to the obtained spectrum.

As mentioned, processing QCDs in the 45°-facet double-pass geometry allows for easy comparison to QCDs in literature, see Table 1. Besides the common InGaAs/InAlAs material system on InP, QCDs have also been grown in other material systems, especially to expand the designable wavelength range to the NIR region [10]. For this reason, In_0.53_Ga_0.47_As/AlAs_0.56_Sb_0.44_ [36], and InAs/AlAs_0.16_Sb_0.84_ [8, 23] were demonstrated. Devices belonging to the shortest wavelength QCDs were realized in GaN-based material systems [37]. However, those material systems with high CBO are less developed and QCDs usually reach lower responsivity.

**Table 1:** Table of selected QCDs from literature, processed in the 45°-facet double-pass method, and the room temperature characteristics: quantum well effective electron mass $m_{e}^{*}$ , wavelength *λ*, peak responsivity **R**_**p**_, and detectivity **D**.

Authors	Material system	Well $m_{e}^{*}$	*λ* (µm)	R_p_ (mA/W)	D (Jones)
Vardi [37]	GaN/AlGaN/AlN on sapphire	0.2	1.7	10	–
Giorgetta [36]	In_0.53_Ga_0.47_As/AlAs_0.56_Sb_0.44_ on InP	0.043	2.46	2.57	1.2 × 10^8^
Giorgetta [10]	In_0.61_Ga_0.39_As/In_0.43Al_Al_0.55_As on InP	0.043	4.05	6	5 × 10^7^
Harrer [20]	In_0.53_Ga_0.47_As/In_0.52_Al_0.48_As on InP	0.043	4.3	10	5 × 10^7^
Dougakiuchi [38]	In_0.53_Ga_0.47_As/In_0.52_Al_0.48_As on InP	0.043	5.4	22	1.1 × 10^8^
Reininger [39]	In_0.53_Ga_0.47_As/In_0.52_Al_0.48_As on InP	0.043	8	16.9	2.9 × 10^7^
Reininger [23]	InAs/AlAs_0.16_Sb_0.84_ on InAs	0.026	4.84	1.9	2.7 × 10^7^

## 4 Conclusions

The growth analysis and optical characterization of four InAs/AlSb QCD designs ranging from 3.65 to 5.5 µm strain balanced with submonolayer InSb layers on GaSb are reported. The growth analysis, including HR-XRD *ω*-2*θ* scans, HR-XRD reciprocal space maps, and AFM surface profiles, indicates excellent strain balancing, no mosaic spread or growth rate fluctuations, and low interface roughness for all four QCDs. Optical characterization with an FTIR and an unpolarized broadband Globar source show peak room-temperature responsivities at the designed QCD transitions. The optimized QCD design at 4.3 µm exhibits a room-temperature responsivity of 26.12 mA/W and a detectivity of 1.41 × 10^8^ Jones. Due to the band alignment of InAs/AlSb and the low band gap of InAs, strong interband transitions in the MIR to NIR range are possible and observed in the QCDs transition spectra, between 1.8 and 3 µm.

## List of Nonstandard Abbreviations

AFM: atomic force microscopy
CBO: conduction band offset
FTIR: Fourier-transform infrared spectroscopy
HR-XRD: high-resolution X-ray diffraction
I–V: current–voltage
MIR: mid-infrared
NIR: near-infrared
QCD: quantum cascade detector
QWIP: quantum well infrared photodetector
TE: transverse electric
TM: transverse magnetic
